# Being black in a white skin: Beliefs and stereotypes around albinism at a South African university

**DOI:** 10.4102/ajod.v4i1.106

**Published:** 2015-05-22

**Authors:** Relebohile Phatoli, Nontembeko Bila, Eleanor Ross

**Affiliations:** 1 Department of Social Development, Gauteng Government, South Africa; 2 Department of Criminology and Social Work, University of Pretoria, South Africa; 3 Centre for Social Development in Africa, University of Johannesburg, South Africa

## Abstract

**Background:** Partly because of the legacy of apartheid, and despite being a constitutional democracy, South Africa continues to be a deeply divided society, particularly along racial lines. In this context many people with albinism do not fit neatly into black and white categories and are likely to experience social discrimination and marginalisation.

**Objectives:** The study endeavoured to explore the beliefs and practices regarding albinism within a South African university, and the availability of support services.

**Method:** The research was located within an interpretive qualitative paradigm and was framed within the theories of stigma, discrimination and ‘othering’. Interviews were conducted with five students with albinism and 10 students without albinism.

**Results:** Findings confirmed the existence of myths and stereotypes regarding albinism. Students with albinism tended to exclude themselves from the rest of the student community to avoid discrimination and stereotypes around their condition.

**Conclusion:** People with albinism can teach us about social constructions of race, colour and relations between minority groups and the majority culture. Results have implications for schools, disability units at universities, and albinism societies in terms of opening up channels of communication between people with albinism and the general public and fostering knowledge and awareness thereof.

## Introduction

The term albinism is derived from the Latin *albus* meaning white. According to Nasr (2010), albinism is a genetically inherited, autosomal recessive physical condition where there is a shortage or absence of the melanin pigment which affects the eyes, hair and skin. People living with albinism are particularly vulnerable to skin cancer and a range of eye problems that can lead to blindness. However, these complications may be prevented by wearing dark glasses and appropriate clothing and by lifelong use of an anti-actinic skin cream (Lund & Gaigher 2002). Estimates of the prevalence of albinism in Africa range from one in 5000 to one in 15 000 with approximately one in 4000 individuals in South Africa. This condition occurs more frequently amongst black people than amongst other population groups (Hong, Zeeb & Repacholi 2006).

### Problem statement

Although most people with albinism tend to have light hair, skin and eyes, their other facial features and hair texture resemble those of Africans and they are usually born into black African families; hence they tend to identify with the black rather than white community. Nevertheless, because of their differences they do not fit neatly into either the black or white groups and tend to be subject to various myths and stereotypes. Although Mohamed Adhikari (2005) was focusing on the South African coloured community rather than persons with albinism when he wrote the book *Not white enough, not black enough: Racial identity in the South African coloured community, this phrase* would seem to encapsulate the contradictions, ambiguities and complexities of albinism.

#### Key focus

Stigmatisation and ‘othering’ of people with albinism is particularly salient in South Africa given its long history of separate development, power differences and institutionalised racism, and the continued existence of these divisions. Despite being a constitutional democracy, post-apartheid South Africa ‘… is still very much a divided society with new kinds of cleavages within and between different ethnic and racial population groups’ (Terreblanche 2012:15). This article focuses on the experience and contradiction of being a black person in a white skin within such a divided context.

## Background

People with albinism face teasing, staring, and well-meaning but ignorant questions about their condition (Nasr 2010). Anecdotal evidence from Nasr (2010) indicates that when people see an individual with albinism, they often make comments like, ‘… the lightning struck his mother during pregnancy and this enables him to read people's thoughts’. In 2012 the director of the Albinism Society of Southern Africa reported:

When we get into taxis, people still move to the other side, or even refuse to use that taxi. We are still called *isishawa* (a Zulu word for a person who is cursed) and *inkawu* [an Nguni word for ‘white baboon’]. (Fazel 2012)

In the folktales of some cultures people with albinism are portrayed as cannibals whilst in Zimbabwe it is thought that having sex with a woman with albinism will cure a man of HIV.

Ntinda (2008) states that in some parts of Africa, people with albinism are perceived as ‘sacrificial lambs’, from the biblical metaphor for someone or something that is sacrificed for the good of others. In the case of these individuals, they are wanted for their hands or genitals which are considered to be the body's strongest parts. People with albinism are hunted for their body parts, as they are believed by some to possess supernatural or magical powers. If you are married to an albino it is believed that you will be a very lucky man or woman.

According to De Groot (2010), people with albinism in Tanzania, Kenya, Zimbabwe and other parts of Africa experience threats to their safety as a result of superstitions which thrive in times of economic deprivation. In response to the widespread disappearance and killing of people with albinism in Tanzania, Burundi, and other East and Central African countries, the United Nations officially declared people with albinism ‘persons with disabilities’ in 2008 (Fazel 2012). As a signatory to the United Nations Convention on the Rights of Persons with Disabilities, South Africa has undertaken to take into consideration the protection and promotion of the human rights of persons with disabilities in all policies and programmes.

The Convention recognises that disability results from the interaction of persons with impairments and attitudinal and environmental barriers that prevent their full and effective participation in society. It further acknowledges the importance of influencing policies at the national, regional and international levels in order to equalise opportunities for persons with disabilities and to promote and protect their human rights. At the same time, the document acknowledges that despite these various undertakings, persons with disabilities such as albinism continue to confront barriers to full participation as equal members of society and experience violations of their human rights.

### Trends

The continued existence of discriminatory beliefs and practices in relation to albinism can be partly explained in terms of the theories of ‘othering’ and stigma. Nevertheless, despite the prevalence of stigma and discrimination, many people with albinism have succeeded in a variety of fields and occupations. Moreover, a study conducted at the University of Venda, in Limpopo, South Africa, found that students with albinism could participate in mainstream education with appropriate intervention to help them manage the problems associated with their low vision and sensitive skins (Mashau 2012).

### Objectives

Given this backdrop, the main aim of this pilot study was to investigate the attitudes of two groups of South African university students concerning albinism, those with and those without albinism. Specific objectives were: to explore the knowledge and understanding of albinism; friendships between students with and without albinism; beliefs and stereotypes and their impact on the self-image and confidence of persons with albinism; and challenges confronting university students with albinism and support services available to them.

### Contribution to the field

According to Braathen and Ingstad (2006), there is a paucity of empirical research undertaken on this condition; hence it was anticipated that this research would add to the current knowledge of albinism and might help conscientise professionals about the challenges confronting people with this condition. It was also envisaged that the research might assist disability units at various universities to formulate policies designed to support students with albinism.

### Literature review

Sociologists have developed a theory of ‘othering’ to describe the processes whereby people who are different tend to be perceived as increasingly alien and distanced (Cromer 2001). This distancing and alienation leads to the emergence of a dividing wall of hostility and suspicion between the insider and outsider, with those on the outside being perceived as ‘others’ and less desirable than ‘insiders’. Some theorists regard othering as a key dynamic underpinning stigma (Deacon, Prosalendis & Stephney 2005).

As early as 1963, Goffman developed what has become the benchmark social theory of the association between stigma and disease. Goffman (1963:13) described stigma as ‘… an attribute that is deeply discrediting, and is socially constructed on the basis of what society regards as being different or deviant’ (Green 2009). In a similar vein, Hawkesworth (2001) indicates that the faces of people with albinism are visually affected by their condition. Nothing is more visible than the face and to be defined by society as facially disfigured, indicates an aesthetic aversion towards those who have a different appearance or deviate from specific conceptions of the so-called ‘normal’ body. Goffman (1963) also referred to the association between stigma and a ‘spoiled identity’. It is therefore of interest that Wan (2003) in her study of 12 persons with albinism, emphasised that the so-called ‘normals’ were the ‘identity spoilers’ or cause of the problem.

For example, Lund and Gaigher (2002) found that respondents in their study felt that people with albinism had fewer friends than people without albinism, and experienced difficulties in mixing at social gatherings. According to Deal (2007), negative beliefs and attitudes which make people with disabilities feel devalued or insecure are likely to influence the individual's psychological well-being and can create subtle forms of self-oppression. In addition, Deal (2007) refers to aversive disablism which denotes discriminatory, oppressive or abusive behaviour arising from the belief that people with disabilities are inferior to others. Moreover, with aversive disablism people with disabilities tend to favour the able-bodied, which may exacerbate the negative emotional effects experienced by people with disabilities (Deal 2007).

Campbell (2008) refers to internalised ableism whereby people assimilate the norm that requires persons with a disability to embrace or assume an identity other than their own. ‘Moreover, internal oppression is not the cause of people with disabilities’ mistreatment but is the result of their mistreatments. It would not exist without the real external oppression that forms the social climate in which people with disabilities exist’ (Mason, cited in Marks 1999:25). Once oppression has been internalised, Marks (1999) believes that people with disabilities harbour the pain, memories, fears, confusions, negative self-images and low expectations, turning these into weapons with which to re-injure themselves.

Herek has endeavoured to build on the earlier work of Goffman and has published extensively on the concept of stigma. Although his work focusses predominantly on stigma in relation to homophobia and AIDS, many of his ideas would seem to be applicable to albinism. He discusses the various dimensions on which different stigmas can be ordered. These include:

- Concealability (the extent to which the stigmatised condition is hidden or obvious).
- Disruptiveness or obtrusiveness (the extent to which it interferes with the normal flow of interaction).
- Aesthetic qualities (whether the condition is repellent, ugly or upsetting).
- The origin of the condition including the affected person's perceived responsibility for its cause or maintenance.
- The course of the stigma (whether the condition is unalterable or degenerative).
- The peril posed by the condition (the extent to which people believe they can be physically, socially or morally tainted by interaction with the affected person (Herek 1990).

These psycho-social dimensions influence how stigmatising conditions are socially constructed. According to Herek (1990), cultural images develop of individual persons as well as communities of affected individuals and are grounded in attitudes and prejudices. These cultural constructions often give rise to competing moral and pragmatic viewpoints. Moralists endeavour to define the condition as a manifestation of spiritual or supernatural forces with the condition being perceived as a punishment or test from God. In contrast, pragmatists tend to look for the scientific explanation for the condition. These world views influence the societal responses to the condition and whether affected individuals are ostracised or treated with compassion. Herek (1990) explains that individual manifestations of stigma represent the intersection of psychological processes with the cultural construction of the condition.

More recently, Herek distinguished between four types of stigma. *Enacted* or *external* stigma refers to negative actions against minorities, such as shunning, ostracism, moral judgement, overt discrimination, and abuse. *Felt* stigma refers to the expectations about the circumstances in which the stigma will be enacted. *Internalised* or *self* stigma refers to the personal acceptance of negative societal attitudes and attendant fear and vigilance as part of one's value system or self-concept. *Courtesy* or *associative* stigma includes family members, health workers, informal carers, and volunteers working in albinism organisations who are not necessarily directly affected by albinism (Herek 2014).

Levels of stigma are also influenced by the levels of knowledge and understanding that people have about the condition, as well as pre-existing attitudes to other groups, for example attitudes towards persons of other ethnic or racial groups (Herek 1990, 2014) and are particularly salient in South Africa with its history of apartheid and divisions along racial lines.

The aforementioned concepts and theories served as the theoretical framework under-pinning the study objectives. Although previous studies on albinism have drawn upon these theories, there is a paucity of research focussing on the lived experience of what it is like to be a black person with a white skin in South Africa. Hence it was envisaged that the present study would fill part of this gap in the research literature.

## Research method and design

### Materials

The research focused on two different samples of participants recruited through non-probability sampling. The first sample comprised students with albinism studying at a South African university. The second sample comprised students without albinism attending the same university. The research tools included two different interview schedules – one for the students with albinism and one for those without.

### Setting

At the time that the study was undertaken, the first author was a student without albinism at a South African university who was interested in the views of her fellow students regarding this condition. Interviews were conducted on the campus.

### Design

This pilot study was located within a qualitative research paradigm. According to Williams *et al*. (2011:53), ‘… the qualitative research approach is based on the interpretive perspective, which states that reality is defined by the research participants’ interpretations of their own realities’.

### Data collection procedure

Data were collected via semi-structured interviews for the students with albinism and for those without. Initially the plan was to interview 10 persons from each group. However, because of the limited number of albino students, it was only possible to recruit five persons with albinism for participation in the study.

### Recruitment procedures

Participants with albinism were recruited through non-probability purposive sampling. A purposive sample is purposively selected by the researcher based on the researcher's judgement (Babbie 2013). The first author consulted with the director of the Disability Unit at the university who allowed her to put up posters to inform students with albinism about the study. The other sampling procedure utilised for the students with albinism was snowball sampling, a technique that begins with a few relevant participants and extends participation through referrals (Babbie 2013).

Non-probability availability sampling was utilised to recruit students without albinism. The researcher approached people at the student centre and those sitting on the library lawns and invited them to participate in the study. The weakness of this sampling procedure is that it lacks scientific rigour and does not allow for generalisation of findings. The strength of this sampling procedure is that it is easy and inexpensive to conduct (Terre Blanche, Durrheim & Painter 2006). However, there was minimal interest in participation amongst students without albinism and it is possible that some students might have been reluctant to reveal their lack of knowledge regarding the condition.

## Analysis

The process of thematic content analysis was utilised to analyse data from the interviews. Content analysis is defined by Grinnell and Unrau (2011:561) as ‘… a data collection method in which communications are analyzed in a systematic, objective, and quantitative manner to produce new data’. The process of data analysis followed the steps recommended by Terre Blanche *et al*. (2006) namely familiarisation and immersion, inducing themes, coding, elaboration, and interpretation and checking.

### Ethical considerations

Permission was granted by the Disability Unit of the university to conduct the study and ethical clearance was obtained from the university's non-medical ethics committee. Indirect benefits included the enhancement of knowledge gained from the study. In order to prevent harm, participants’ privacy and confidentiality was respected and counselling was offered to participants experiencing distress. Potential participants were assured that participation was voluntary and that they had the right to withdraw from the study without negative consequences. Participants were given an information sheet explaining the purpose and procedures of the study and their rights as research participants. Thereafter, they signed informed consent forms. All information was kept confidential and no identifying details were included in the final report. They were assured that all raw data would be kept in a locked cupboard for two years following any publications and for five years if no publications emanated from the study. Thereafter all raw data would be destroyed.

### Trustworthiness

In order to enhance validity and reliability of data collection a pre-test of the research tools was conducted with one student with albinism and one without who were excluded from the final study. Based on recommendations from the pre-test, both interview schedules were amended accordingly. All interviews were conducted by the first author.

In order to further enhance the trustworthiness of the data, the four constructs of credibility, dependability, confirmability and transferability were taken into consideration (Trochim 2006). The researchers endeavoured to enhance *credibility* or plausibility of the study by providing a detailed theoretical framework, aligning the questionnaires with the theoretical framework and by pre-testing the research tool. In order to enhance *dependability* or replicability of the study the same questionnaires were given to all the participants by the same researcher. However, the use of a small, non-probability sample precluded *transferability* or generalisation of the findings to the broader population of persons with albinism or university students. In terms of *confirmability,* an objective independent colleague checked the categorisation of themes presented by the primary researcher for correspondence. After reaching agreement on the themes, these were quantified.

## Results

Results are presented in accordance with three areas of analysis highlighted by Herek (1990), namely, the subjective experience of persons with albinism, the knowledge and attitudes towards albinism of persons without albinism, and the interaction processes through which the two groups negotiate their respective roles in social interaction. Thereafter, the view of both groups are presented regarding the way forward.

**TABLE 1:** Profile of participants (*N* = 15).

Profile	Demographic factor	Sub-category	Number
**Persons with albinism (*n* = 5)**	Gender	Male	2
		Female	3
	Age range	20–26 years	
	Level of study	2nd	2
		3rd	1
		4th	2
**Persons without albinism (*n* = 10)**	Gender	Male	2
		Female	8
	Age range	19–31 years	
	Level of study	1st	3
		2nd	3
		3rd	4

### Description of participants

#### Perspectives of people with albinism

For participants with albinism, understanding of the condition was based on how it had affected their lives and their own personal experiences with the condition. Three of the five participants understood the condition of albinism based on medical aspects. This theme was captured in the following statement: ‘For me albinism is a hereditary condition characterised by the complete or partial absence of pigment in the hair, skin and eyes’.

In contrast, two out of the five participants expressed their understanding and knowledge of the condition of albinism based on how the environment had treated them and how they were perceived by others. This theme was captured in the following verbatim quote:

‘Honestly, I think albinism is a curse because people always stare at me, talk about me behind my back and also make very nasty remarks when I pass, saying that I do not know that my father is not black but is a white person and that is why I look the way I do’.

Participants living with albinism reported having experienced discrimination from people without albinism. They felt that they were seen as outcasts and most of the time they had to prove that they were normal people and that they could do things and achieve as well as people without albinism. This was encapsulated in the following statement:

‘It has been extremely difficult to live with albinism. I always have to prove myself, work extra hard and put in more effort into everything so that people can see me as ‘’normal”’.

Another participant explained:

‘I always thought that for me it was normal, but sometimes I find myself in a situation whereby I have to explain my condition to other people so that they see me as normal and as one of them. So when I think of it, it was kind of hard because people without albinism are not treated the way we are treated’.

The participants informed the researcher that their friends and neighbours or people who knew them treated them well and made them feel comfortable and normal. However, with strangers it was difficult and they did not like being in such environments because people tended to stare at them. This theme was reflected in the following statements: ‘Once people get to know me then they relax and see me as a normal person but for people who don’t know me they stare at me’*.* Racial stereotyping was evident in the following response:

‘People don’t treat me the same. There are some who do not treat me normally and are always staring at me and they say bad things about me, things like maybe my father is not really my father, or maybe my mother had an affair with a white man’.

Participants with albinism also reported being aware of beliefs regarding it. One participant mentioned that he had heard that in Uganda they chopped off the heads of people with albinism and used these body parts for *muti* [traditional medicine]. Another participant stated that she had been told that when people with albinism died they turned into salt and they were only buried at night and not during the day like everyone else. The remaining three participants had heard stories about using bones from people with albinism for good luck and *muti*, as well as identifying them as a curse on the family and a punishment from God.

Three out of the five participants with albinism appeared to have been negatively affected by the stereotypes and beliefs around albinism as reflected in the following statement:

‘These beliefs and stereotypes have affected me because as a child other children always used to ask if they are true or not and because of that I have a low self confidence, and other people don’t see me as normal’.

However, the remaining two participants reported that these stereotypes did not exert much of an effect because of the support from their families and loved ones.

Three out of the five participants reported experiencing some childhood challenges because of the people in their communities. For one participant, childhood was not affected in any way. It was only when she was older that she understood what was said about her so that she had to take extra care of herself just to prove that she was normal. However, for the other participant, growing up was not difficult because of the confidence that she had gained from the support of family and friends.

### Perspectives of people without albinism

Three of the ten participants without albinism showed no knowledge and understanding of the condition, as encapsulated in this response:

‘No I don’t know anything about albinism, I just know that it is people who are too light and have strange eyes because most of the time their eyes are rolling around and they don’t keep still’.

Five of the participants without albinism showed some knowledge without fully understanding the condition, having heard about albinism through the media and from other people. For example:

‘Honestly, I have little knowledge about albinism. I just know that they have a skin problem, different from our skin and they should avoid too much exposure to the sun’.

‘I know that they are stigmatised and people do not have much knowledge about albinism and their genes, they relate to them to be a curse and culturally they are stigmatised in the communities. People like me do not get time to know about their biological genetic formations’.

The remaining two participants without albinism showed in-depth knowledge of the condition from the subject, Life Orientation, which had been taught at primary and high school and from their relationships with people with albinism. One of them stated that as a result of constant interaction with a person with albinism:

‘I had to learn about this condition and that helped so that I could see him as a person like me and not discriminate against him, even though there were some stigmas around albinism, they did not change my views about my friend because I know about his condition’.

The other participant also shared her understanding of albinism by stating:

‘It is a skin condition that is related to pigmentation. It is a lack of melanin, and if you have a lot of it you are darker and if you have a small amount you are lighter. Albinos do not have it and for me I see it as more of a skin condition rather that a disease. People with albinism often have eye problems and that shortage of the pigment is also present in the hair and that makes their hair colour to become lighter or blonde’.

From the study it emerged that 9 of the 10 participants without albinism did not have any relatives with albinism and only two participants reported having friends with albinism. The one participant explained his relationship with a few people in his life who had albinism as follows:

‘I have people who are very close to me, whom I have considered them not only as my friends but as relatives. Like my pastor at church, he is a father figure to me and I look up to him, and I also have a friend whom I consider to be my brother because we are very close. At res in primary and high school where I was he used to be my best friend, we used to share food, share a bed and we also did everything together, even study together and that is how he became a brother to me’.

The other participant reported that she had never had an albino friend or relative but her mother used to have a close friend with albinism at nursing school. Consequently, she developed an interest in learning about the condition because when her mother told her about her friend she had emphasised the latter's beauty and generosity.

When the participants without albinism were asked if they would date a person with albinism, 6 of the 10 participants said they would not do so. This theme was reflected in the following quotes: ‘I will be scared of intimacy with them’. One student commented:

‘I don’t think I would because I am scared at the way they look at people. Their eyes are always moving and sometimes they can’t stay in one position. I would also be scared of having a child with albinism’.

Another participant reflected: ‘I don’t have a reason why I would not but I just don’t think I would’.

Only one participant stated that she would be prepared to have a relationship with a person with albinism. She commented:

‘I am not stereotyped. I would judge a person by their attitudes and their behaviour. Other albinos are cute and look very handsome with good physical structures, however, their attitudes will determine if I would date them or not’.

The remaining three participants had mixed emotions about dating people with albinism. For example:

‘I am not opposed to it, and since people with albinism are such a minority I think there are slim chances that I would find a person with albinism whom I like and get along with. I also have my own stereotypes about albinism and that would make it a challenge’*.*

Another participant expressed the influence of religious views as follows: ‘If the Lord says so then I would because he is the one who is directing my love life at the moment’. The influence of cultural beliefs and practices was captured in the following response:

‘It is a very difficult question, but I don’t have anything against albinos. I regard them as normal people. My experience and beliefs will not deny me the opportunity, however, in my culture [*African culture*] you don’t get married only for yourself but it is for the whole family, so it will be very difficult to convince them and educate them about albinism’.

When the participants were asked if they would make friends with people with albinism, 9 of the 10 replied in the affirmative, based on the belief that they were human beings just like everyone else; however, they would not have intimate relations with them. One participant indicated the influence of myths and stereotypes when he mentioned the reasons why he would not make friends with them: ‘I was told they contain bad luck and even when I was very young I was not friends with a boy in our street because he was an albino’.

It emerged that all 10 participants without albinism were aware of beliefs and stereotypes regarding albinism as reflected in the following quotes:

‘Albinos don’t die, they just go missing and they disappear. Last year when I was pregnant, I was told that I must not look at the albinos because if I do I will get a child with albinism or if I do look at them by mistake I should spit at them to avoid a child with albinism’.

‘When I was growing up I was told that albinos are ugly and they are not physically attractive, I also remember that they used to say that children with albinism are the results of their parents’ evil doing so they were considered as a curse in the community’.

‘From where I come from there are very few albinos, but I have worked in Tanzania, Congo, and in Zimbabwe. In Zimbabwe they believe that if you are HIV/AIDS positive and you sleep with an albino you will get cured from the disease. In Tanzania, they believe that albinos have magical powers. If you kill an albino and you have a bone you can get rich. The private part of an albino, the blood, hair and flesh will make you rich and help you get a promotion at work. I also remember a testimony of a woman from Tanzania, who is the first woman with albinism in parliament. This woman said that when she was growing up people would refer to her as a ghost and her friend's parents would not let her friends play with her or even share a bed with her because then her friends’ mothers would have babies with albinism’.

‘In my community they still believe that when you pass an open fire you should spit in your own shirt because if you don’t then it will bring you the bad luck of having a child with albinism in the future. This only applies to females and girls of all ages but mostly to girls who don’t have children yet. They used to say a child who is an albino brings bad luck into the family because they are a result of witchcraft so they use them for muti’.

### The interaction between the participants with albinism and those without albinism

Nine of the ten participants without albinism reported having no relationships with students with albinism. They attributed this to the fact that there were only a few students with albinism at the university where the study was conducted and hence they did not attend the same lecture courses or move in the same circle of friends with them. This theme was captured in the following statement:

‘I see them around but I don’t have any personal relations with them. They are very few and they usually exclude themselves from the rest of the other students. I don’t see them very often and when I do see them they are often by themselves or with their usual small group of friend’.

One participant had a relationship with a student with albinism because they were in the same student residence. They had initially started their relationship by greeting each other, and had subsequently become mutual friends.

Of the five participants with albinism who participated in the study, three reported negative experiences of being a university student. They felt that because of other students’ ignorance and lack of knowledge about the condition, they sometimes had to defend themselves and always remind themselves that they were ‘normal’ persons. In class it was sometimes difficult for them to read lecture notes and writing on the board, and they did not want to continually depend on the other students.

However, the remaining two participants acknowledged that if they did not know people and if they were not confident then it was hard for them. However, it helped if they were confident and they avoided unfamiliar environments where their friends were not present. Challenges included fitting in with the rest of the population and other students, keeping up in lectures because of the difficulty of reading lecture notes, being excluded by other students and other students staring at them and talking about them behind their backs.

It emerged that the social environment played an important role in shaping their behaviour. The participants stated that at school, if the other students treated them badly and teased them about their skin colour and their eyes, they would have negative thoughts and emotions about who they were. However, because their families had been supportive, they had succeeded in coming to university, following their chosen careers, and making sure that they did what ‘normal’ people do. They reported that the Careers, Counseling and Development Unit (CCDU) and the Disability Unit (DU) at the university concerned had been the best social support systems in their environment.

However, some people in the participants’ social environment related to them in the way they did because of their condition. For example, their family and friends would always offer to share things like an umbrella or skin protection lotions without considering that they had the means to take care of their skin.

Nine of the ten participants without albinism reported that they found it easier to make friends with students who did not have albinism, because there were far more students without albinism at the university and the students with albinism tended to distance and exclude themselves from the other students. However, one participant stated that it was taboo to have a friend with albinism and because of the stereotypes around albinism: people would stare at you if you had a friend with albinism.

Three participants with albinism found it easier to make friends without albinism than ones with albinism at the university because of the minority of the students with albinism. These participants stated that they saw themselves as normal people rather than just people with albinism. The one participant had no problem with either students with or without albinism. In her view people were just people no matter their condition.

### Views of both groups regarding the way forward

The 10 participants without albinism felt that it was important for them to know more about the condition and to know how people with albinism deal with the stereotypes about it. One participant stated that from the medical side he wished to know about the genetic factors underpinning albinism so that he could understand more about their skin pigmentation and to find out what went wrong with their genes. Furthermore, he wanted to learn about traditional healers’ views on the causes of albinism and why they believe that it only comes once in each generation of a family.

Participants with albinism believed that social workers had a role to play in assisting all students with albinism at university. It emerged that the students with albinism felt that social work services could be most valuable to first year students with albinism, especially during orientation week because it would help new students to socialise better, boost their self-confidence and gain social support. It was suggested that social workers needed to facilitate support groups and education programmes for both students with and without albinism, because students who wanted to know about the condition could have open discussions about it with students with albinism. Such activities would afford the latter opportunities to interact with more people without albinism and have an open platform to discuss the stereotypes and acquire more knowledge. Furthermore, such an approach could potentially enhance their sense of agency and thereby achieve social change.

Participants with albinism stated that they wanted to be treated like normal people and for others to not treat them like they had a disease. They expressed their concerns by stating:

‘People need to know that we have feelings and that we are normal just like them. It is important for them to know that their stereotypes affect us and they hurt us because we do not know what we did in life to deserve such criticism from them. We also deserve to be treated with respect’.

‘People should just realise that we are normal people just like them and that sometimes it is difficult to deal with all those stereotypes and beliefs they have about us or albinism in general because even when they are not talking about you, you immediately assume that are’.

## Discussion

Findings from the present study suggested that several of the participants lacked knowledge regarding albinism, which may be attributed in part to the fact that very few participants had relatives or friends with albinism. Similar results were obtained by Lund and Gaigher (2001) who conducted a study in the Capricorn district, Limpopo, South Africa, and found that 28 out of 38 participants did not know what caused albinism. Braathen and Ingstad (2006) also found that many of the 25 people with albinism and their family members whom they had interviewed in Malawi had very little knowledge about the condition although they were aware of the need to take precautions to prevent excessive sun exposure.

Persons with albinism found it difficult to interact with strangers although friends and relatives treated them well. The fact that they tend to avoid interactions with the broader student body suggests that they experienced the type of alienation highlighted by Fanon (1967) in his classic text *Black skin*, *white masks.* Persons without albinism indicated their willingness to befriend persons with albinism whilst not necessarily going on dates with them. However, the limited number of students with albinism restricted opportunities for socialising.

All the participants with albinism had experienced the discrimination and stigma described by Goffman (1963) and Herek (1990; 2014). Results suggested the existence of both enacted and perceived stigma in relation to persons with Albinism. The fact that one participant reported having ‘… spat upon a person with albinism’ is a blatant example of enacted stigma (Herek 2014). Both these forms of stigma may have severe social consequences for people in terms of their rights, self-identity, freedom and social interaction, and they may have far-reaching psychopathological ramifications for the affected person. In this respect, Herek (1990) maintains that stigma may affect an individual's self-concept and self-esteem.

Some participants with albinism tended to adopt the coping mechanism of excluding themselves from the rest of the student population in order not to be judged or discriminated against. According to Herek (2014), perceived stigma relates to feelings of shame and the oppressive fear of enacted stigma and predisposes the stigmatised persons to avoid exposing their condition to protect themselves from experiencing discrimination. It is characterised by self-exclusion from services, alienation and social withdrawal, loss of identity, poor self-image, and overcompensation. Such social isolation strategies often have negative psychological consequences such as depression and anxiety (Herek 2014).

Findings also alluded to the use of labelling, stereotypes, and ‘othering’ in relation to persons with albinism. According to Green (2009), labelling occurs where human differences are noted and labelled; stereotyping, where labels are imbued with negative stereotypes; and othering, where labelled persons are categorised as ‘other’ or ‘them’ in order to clearly separate ‘them’ from ‘us’.

In line with Herek's (1990) theory on stigma, it was also evident that culture exerted a significant influence on the understanding of students without albinism regarding the phenomenon under discussion. In this regard, Ntinda (2008) postulates that albinism is surrounded by many cultural beliefs, superstitions and stereotypes especially in Africa with its diverse cultures. For example, the perception that albinism was caused by a black woman sleeping with a white man, highlights the intersection of race, colour and gender in the social construction of such beliefs. The view that albinism was caused by the parents’ evil actions was consistent with Fanon's (1967) idea of projection in his discussion of psychopathology, and Herek's (1990) notion of assigning blame for the condition. Similarly, the fear of having a baby with albinism if one associated with such a person during pregnancy was in line with Herek's notion of peril and contamination. The fact that some participants were prepared to befriend persons with albinism but would nevertheless not want to go on dates with them, illustrated the need to maintain a degree of social distance. These negative cultural beliefs in turn affected the self-confidence of persons with albinism. Their need to work harder to overcome these stereotypes suggests the use of the defence mechanism of over-compensation emphasised by Fanon (1967).

Participants without albinism referred to the role of the media in influencing their attitudes towards albinism. In this respect, Hawkesworth (2001) affirms that the stereotypes, visual images and jokes about facial disfigurement often occur in the mass media. In the study conducted by Hawkesworth (2001) the respondents believed that the media influenced responses to facial difference.

Challenges included difficulty fitting in, coping with ignorance, being stared at, and problems associated with reading lecture notes and lecturers’ writing on the board. Nevertheless, they were able to derive support from families, friends and the Disability Unit at the university.

### Limitations of the study

The limitation of using non-probability sampling is that it precluded generalisation of the findings to the broader population of persons with albinism. Moreover, there were only a limited number of students with albinism, several of whom were reluctant to participate in the study. Hence the study needs to be replicated on a larger, more representative sample. The fact that the researcher did not have albinism may also have influenced participants’ responses. In addition, the sensitive nature of some questions may have resulted in the furnishing of socially desirable responses.

## Conclusions and recommendations

Three main conclusions could be drawn from this study. Firstly, the stereotypes, beliefs and lack of knowledge around albinism dramatically affected the way people without albinism interacted with people with albinism which in turn influenced the way people with albinism in the study viewed themselves and how they needed to present themselves to prove their worth. Secondly, participants with albinism tended to exclude themselves from the rest of the population in order not to be judged or discriminated against. According to Herek (2014) such social isolation strategies often have negative psychological consequences. Perceived stigma on the other hand relates to feelings of shame and the oppressive fear of enacted stigma and predisposes the stigmatised persons to avoid exposing their condition to protect themselves from experiencing discrimination (Green 2009). It is characterised by self-exclusion from services, alienation and social withdrawal, loss of identity, poor self-image, and overcompensation. Thirdly, those persons who had become friends with people with albinism were able to value and appreciate the person behind the condition.

From the research responses, it emerged that some schools provide knowledge and awareness programmes about the condition of albinism in the Life Orientation curriculum. It is therefore recommended that learners in schools in both rural and urban areas be taught about this condition as part of their syllabus in order to counteract prevailing myths and stereotypes about albinism.

Social workers and other helping professionals need to educate parents, learners, teachers, families, traditional healers and the general public about the aetiology of albinism, effects of discrimination against people with albinism, ways of treating people with the condition and how to handle discrimination as a person with albinism. The media also need to consider the way in which they portray people with albinism (Ekurhuleni declaration on the rights of persons with Albinism 2013)

In addition, there is a need to open up communication channels between people with and those without albinism so that they can share concerns and ways of alleviating problems arising from cultural beliefs and stereotypes. Exposure of the public to persons with albinism can potentially demystify the condition and create positive encounters. For example, Ash *et al.* (1997) found that college students who had attended school with disabled pupils were more likely to have friendships with disabled students at the college level than those who had not had previous exposures. Consequently, people living with albinism can potentially play an active role in breaking the cycle of stigma and discrimination. As Reeve (2002) emphasises, disabled people are not simply passive victims of ‘emotional disablism’. Many exercise agency and resist negative stereotypes.

Counselling and Career Development Units as well as Disability Units at universities and Further Education and Training (FET) colleges need to engage in education and awareness programmes with university students and lecturers regarding the condition of albinism, the impact of beliefs and stereotypes on persons affected by this condition, and the challenges posed by tertiary environments such as reading lecture notes. They need to ‘deconstruct’ albinism by identifying the symbolic images, metaphors and stereotypes surrounding the condition. They also have a responsibility to provide individual counselling and group support programmes to assist students with albinism to integrate more successfully within the university environment. Albinism societies also have a pivotal role to play in advocating for the rights of people with albinism. According to Herek (1990; 2014), such groups help people to understand and overcome their stigmatisation.

In conclusion, the study allowed the voices of a group of persons with albinism to be heard as well as those of their university counterparts without the condition. The message that came across strongly was the need to enhance knowledge and awareness of albinism and thereby create a safer, more just, humane and caring society where the rights of all groups are respected, including those with albinism.
